# Combined Toxicity of TiO_2_ Nanospherical Particles and TiO_2_ Nanotubes to Two Microalgae with Different Morphology

**DOI:** 10.3390/nano10122559

**Published:** 2020-12-20

**Authors:** Zhuang Wang, Shiguang Jin, Fan Zhang, Degao Wang

**Affiliations:** 1 Jiangsu Key Laboratory of Atmospheric Environment Monitoring and Pollution Control, Collaborative Innovation Center of Atmospheric Environment and Equipment Technology, School of Environmental Science and Engineering, Nanjing University of Information Science and Technology, Nanjing 210044, China; jinshiguang12138@163.com (S.J.); zhangfan_nuist@163.com (F.Z.); 2 School of Environmental Science and Technology, Dalian Maritime University, Dalian 116023, China; degaowang@163.com

**Keywords:** TiO_2_ nanoparticles, TiO_2_ nanotubes, nanotoxicity, freshwater algae, oxidative damage

## Abstract

The joint activity of multiple engineered nanoparticles (ENPs) has attracted much attention in recent years. Many previous studies have focused on the combined toxicity of different ENPs with nanostructures of the same dimension. However, the mixture toxicity of multiple ENPs with different dimensions is much less understood. Herein, we investigated the toxicity of the binary mixture of TiO_2_ nanospherical particles (NPs) and TiO_2_ nanotubes (NTs) to two freshwater algae with different morphology, namely, *Scenedesmus obliquus* and *Chlorella pyrenoidosa*. The physicochemical properties, dispersion stability, and the generation of reactive oxygen species (ROS) were determined in the single and binary systems. Classical approaches to assessing mixture toxicity were applied to evaluate and predict the toxicity of the binary mixtures. The results show that the combined toxicity of TiO_2_ NPs and NTs to *S. obliquus* was between the single toxicity of TiO_2_ NTs and NPs, while the combined toxicity to *C. pyrenoidosa* was higher than their single toxicity. Moreover, the toxicity of the binary mixtures to *C. pyrenoidosa* was higher than that to *S. obliquus.* A toxic unit assessment showed that the effects of TiO_2_ NPs and NTs were additive to the algae. The combined toxicity to *S. obliquus* and *C. pyrenoidosa* can be effectively predicted by the concentration addition model and the independent action model, respectively. The mechanism of the toxicity caused by the binary mixtures of TiO_2_ NPs and NTs may be associated with the dispersion stability of the nanoparticles in aquatic media and the ROS-induced oxidative stress effects. Our results may offer a new insight into evaluating and predicting the combined toxicological effects of ENPs with different dimensions and of probing the mechanisms involved in their joint toxicity.

## 1. Introduction

In the past decade, research on the health and environmental risk of engineered nanoparticles (ENPs) has increased considerably [1,2,3]. The requests for toxicity data on the effects of ENPs on organisms continue to grow. Most of the toxicity data are derived from a single toxicity [4,5]. In the natural environment, organisms exposed to a mixture of multiple contaminants (*n* ≥ 2) rather than individual ones is a universal law [6,7]. Many studies have addressed the toxic effects of a mixture of ENPs and other contaminants, e.g., nano-TiO_2_ and tetracycline [8], nano-TiO_2_ and hexavalent chromium [9], as well as nano-TiO_2_ and bisphenol [10]. However, researchers in the field of nanotoxicology have just started to investigate the combined toxic effects of multiple exposures to ENPs.

The existing literature on the combined toxicity of ENPs mainly focus on the modes of joint action (MOJA) [11,12,13,14], which include antagonistic, synergistic, and addition. Moreover, the toxicity effects and MOJA of ENP mixtures are associated with the ENP components and test organisms. Most previous studies have selected ENPs with different components, but their nanostructures have the same dimensions. A few studies have indicated that graphene oxide as a two-dimensional ENP can affect the toxicity of concomitant zinc oxide nanospherical particles (NPs) to aquatic species [15] and human cells [16]. Therefore, toxicological studies on ENPs mixtures are urgently needed to reveal the combined effects induced by ENPs with different dimensional structures.

Nano-TiO_2_ is one of the most promising ENPs [17], and it is described as the “star” of many ENPs. Compared with TiO_2_ NPs with zero-dimension, one-dimensional TiO_2_ nanotubes (NTs) display enhanced excellent properties such as a larger diameter, higher specific surface area, and so on. Thus, the preparation and application of TiO_2_ NTs have provoked great interest among scientific researchers [18,19]. Freshwater algae, like *Scenedesmus obliquus* [20,21] and *Chlorella pyrenoidosa* [22,23] are frequently chosen as a model organism to assess the aquatic toxicity of ENPs, especially as *S. obliquus* and *C. pyrenoidosa* have a different morphology, namely, they are flat- and spherical-shaped, respectively. These characteristics mean that these two algae show a distinct sensitivity to ENPs.

In the present study, we investigated the toxicity of TiO_2_ NPs, TiO_2_ NTs and the binary mixtures of TiO_2_ NPs and NTs, to *S. obliquus* and *C. pyrenoidosa*. The four main objectives were: (1) to determine the physicochemical properties and to evaluate the stability of TiO_2_ NPs, TiO_2_ NTs and TiO_2_ NPs + NTs in a freshwater model; (2) to investigate the growth inhibition toxicity of TiO_2_ NPs, TiO_2_ NTs and the TiO_2_ NPs + NTs to the algae; (3) to assess the MOJA of TiO_2_ NPs and NTs and to predict their joint toxicity using the concentration addition (CA) and independent action (IA) models; and (4) to explore the mechanism of cytotoxicity induced by TiO_2_ NPs, TiO_2_ NTs, and TiO_2_ NPs + NTs by determining the generation of reactive oxygen species (ROS).

## 2. Methods

### 2.1. Test Material and Test Medium

TiO_2_ NPs with a diameter of 21 ± 10 nm (powder, P25 grade) and TiO_2_ NTs with a diameter of 10 nm and a length of 1 μm (wet cake, liquid: water 9.5% wt) were purchased from PlasmaChem GmbH (Berlin, Germany) and Nanjing XFNANO Materials Tech Co. Ltd. (Nanjing, China), respectively. The stock suspensions of the test materials were freshly prepared in ultra-high pure water after 30 min sonication in a water bath sonicator and then stored at 4 °C until use. Algae culture medium was prepared as a test medium at pH 7.8 ± 0.2 according to Organization for Economic Co-operation and Development (OECD) guidelines [24].

### 2.2. Physicochemical Analysis

The morphology of the single and combined test materials in the test medium was characterized by a transmission electron microscope (TEM, JOEL 2100f, JOEL Ltd., Tokyo, Japan). Zeta potential (*ZP*) and hydrodynamic diameters (*HD*) of the particle suspensions at 5 mg/L were analyzed at 0 h and 96 h in the test medium using a ZetaSizer instrument (Nano ZS90, Malvern Instruments Ltd., Worcestershire, UK). The *ZP* and *HD* measurements were performed in three independent experiments, and the data presented are the mean of the runs. Based on classical DLVO theory [25], the stability of a particle in the test medium was determined by simulating the total potential energy for interactions between the TiO_2_ particles.

### 2.3. Algal Growth Assays

The unicellular freshwater algae *S. obliquus* and *C. pyrenoidosa* were obtained from the Chinese Academy of Sciences Institute of Hydrobiology (Wuhan, China). Exponentially growing algae cells (with a final density of 3 × 10^5^ cells/mL for *S. obliquus* and 4 × 10^5^ cells/mL for *C. pyrenoidosa*) were added to the control and treated experiments. Internal control experiments were required to eliminate the absorbance effects of the test materials. All flasks containing various treatments were incubated in an artificial growth chamber at a consistent temperature of 24 ± 1 °C for 96 h with a photoperiod of 12-h light (3000–4000 l×) and 12-h dark. The algal cell density was determined using an ultraviolet-visible spectrophotometer (UV1102; Shanghai Tian Mei Scientific Instrument Co., Shanghai, China) after 96 h for *S. obliquus* and 72 h for *C. pyrenoidosa* to provide cell numbers and allow the specific growth rate to be calculated. Growth inhibition (%) was calculated by dividing the specific growth rate for a treatment by the mean specific growth rate for the controls. Three replicates were included for each treatment and the data presented are the mean of the runs (*n* = 3).

### 2.4. Oxidative Stress Biomarker Assays

2′,7′-dichlorodihydrofluorescein diacetate (DCFH-DA) purchased from Macklin Biochemical Co., Ltd. (Shanghai, China) was used as a fluorescent probe to measure the intracellular ROS. The 96 h (*S. obliquus*) and 72 h (*C. pyrenoidosa*) algal cell suspensions were centrifuged at 15,000 rpm for 10 min at 25 °C (using a D3024 high speed micro-centrifuge (Scilogex, Rocky Hill, CT, USA). After discarding the supernatant, 10 μM DCFH-DA was incubated with algal cells for 30 min under dark conditions at 25 °C. Subsequently, the samples were centrifuged under the same conditions and washed one time with the test medium to remove the loosely bound fluorescent probe. Each test concentration was replicated two times in an independent experiment.

When intracellular ROS are generated, 2′,7′-dichlorofluorescein (DCF) can be converted from DCFH, which is obtained by lipase decomposing DCFH-DA in cells [26]. Thus, the fluorescence intensity (FI) of DCF indicates the extent of intracellular ROS generation. FI was measured using a fluorospectrophotometer (F96PRO, Shanghai Kingdak Scientific Instrument Co., Ltd., Zhejiang, China). The excitation and emission wavelength for the optical measurements were based on previous studies [15,27]. Each sample was measured three times. Data were expressed as a percentage (%) of the fluorescence or the absorbance of the control cells according to the equation:

%*F* = 100 − [100(*F*_c_ − *F*_t_)/*F*_c_] (1)

where %*F* is the percentage of fluorescence of algal cells; *F*_c_ is the mean fluorescence of control cells; and *F*_t_ is the mean fluorescence of treated cells.

### 2.5. Assessment and Prediction for Mixture Toxicity

The logistic model (Equation (2)) was used to fit concentration-response curves (CRCs) for TiO_2_ NPs, TiO_2_ NTs, and TiO_2_ NPs + NTs. The effect concentrations, such as the 10% effect concentration (*EC*_10_) and median effect concentration (*EC*_50_) of each treatment group were derived from the CRCs. The combined toxicity tests followed the mixture ratios of the effect concentrations of TiO_2_ NPs and NTs at the same effect level.

(2) $$E=\frac{100}{\left(1+{\left(\frac{C}{EC_{50}}\right)}^{\theta }\right)}$$

where *C* is the test material’s concentration and *θ* represents the slope parameter.

The *EC*_50_ value was used to calculate a toxic unit (TU), as shown in Equations (3) and (4),

(3) $$\mathrm{TU}=\frac{C_{i}}{EC_{50}}$$

(4) $${\mathrm{TU}}_{\mathrm{Total}}={\mathrm{TU}}_{\mathrm{NPs}}+{\mathrm{TU}}_{\mathrm{NTs}}$$

where *C_i_* is the concentration of material *i* and TU_Total_ is the sum of the TiO_2_ NP and NT TUs. Therefore, one TU corresponded to the *EC*_50_. The MOJA of TiO_2_ NPs and NTs were evaluated by plotting the observed TU (TU_obs_) derived from the toxicity data against the expected TU (TU_exp_), as described by Kim et al. [28].

The total concentration of a mixture provoking an *x*% effect (*EC_xmix_*) was calculated from the CRCs of the individual components using the CA model, as shown in Equation 5.

(5) $$EC_{x\mathrm{mix}}={\left(\sum_{i=1}^{n}\frac{P_{i}}{EC_{xi}}\right)}^{-1}$$

where *P_i_* is the fraction of component *i* in the mixture and *EC_xi_* is the concentration of component *i* that would, when applied singly, provoke the *x*% effect.

The general equation shown in Equation (6) was used for the IA model,

(6) $$E(C_{\mathrm{mix}})=1-\prod_{i=1}^{n}\left(1-E(C_{i})\right)$$

where *E*(*C_mix_*) is the effect expected at the total concentration of the mixture (scaled to between 0% and 100%) and *E*(*C_i_*) is the effect that the *i*th mixture component would provoke if applied singly at concentration *C_i_*.

### 2.6. Statistical Analysis

All data are expressed as means ± standard deviation (SD). Statistically significant differences between the test treatments were determined by a *t*-test at significance levels of *p* < 0.05, *p* < 0.01, and *p* < 0.001.

## 3. Results and Discussion

### 3.1. Physicochemical Characterizations

**Figure 1:**
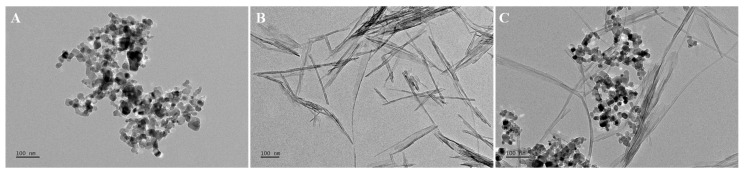
TEM images of the TiO_2_ NPs (**A**), TiO_2_ NTs (**B**), and TiO_2_ NPs + NTs (**C**) in the algae medium.

The TEM images are shown in Figure 1. The individual TiO_2_ NPs and NTs were spherical (Figure 1A) and tubular (Figure 1B) particles, respectively. Additionally, the TEM images show that the TiO_2_ NPs agglomerated heavily in the test medium, whereas the TiO_2_ NTs agglomerated slightly. As can be seen from Figure 1C, the morphology of the two TiO_2_ particles with different shapes in the binary mixture systems was not affected by each other.

To characterize the change in the physicochemical properties of TiO_2_ particles, the *ZP* and *HD* values of TiO_2_ NPs and NTs from single to binary mixtures were measured in the test medium (Table 1). The *ZP* value of TiO_2_ NPs at 96 h were significantly increased compared with the *ZP* value of TiO_2_ NPs at 0 h (*p* < 0.01). It was found that there was no obvious change in the *ZP* value of TiO_2_ NTs or TiO_2_ NPs + NTs between 0 h and 96 h (*p* > 0.05). The measurement of *HD* showed that the of the TiO_2_ particles became smaller in size over a 96-h period. Furthermore, the size of the particles in TiO_2_ NPs + NTs was significantly decreased during 96 h of exposure (*p* < 0.01). The reason for the reduction in particle size may be due to the sedimentation of large particles.

**Figure 2:**
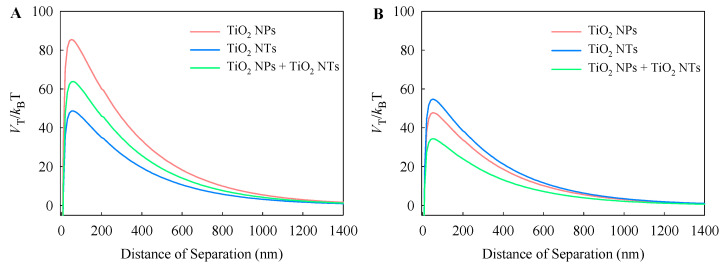
Total potential energy (V_T_) curves for the TiO_2_ particles in the single and binary systems at 0 h (**A**) and 96 h (**B**).

To further evaluate the agglomeration and stability of the TiO_2_ particles in the test medium, the total potential energy profiles of the TiO_2_ particles in the single and binary mixture systems at the end of different intervals were calculated using the DLVO theory (Figure 2). At 0 h, the peak values of the total potential energy profiles decreased in the order: TiO_2_ NPs > TiO_2_ NPs + NTs > TiO_2_ NTs (Figure 2A). This means that the TiO_2_ NPs showed the highest stability in the test medium compared to the other studied systems. However, over 96 h, the stability of TiO_2_ NPs and TiO_2_ NPs + NTs obviously decreased, while the stability of TiO_2_ NTs enhanced slightly (Figure 2B). As mentioned above, the particle size of TiO_2_ particles in the test medium became smaller with time. Taken together, this suggests that the TiO_2_ particles were settling due to particle agglomeration.

### 3.2. Toxicity of Single and Mixtures of TiO_2_ NPs to Algal

**Figure 3:**
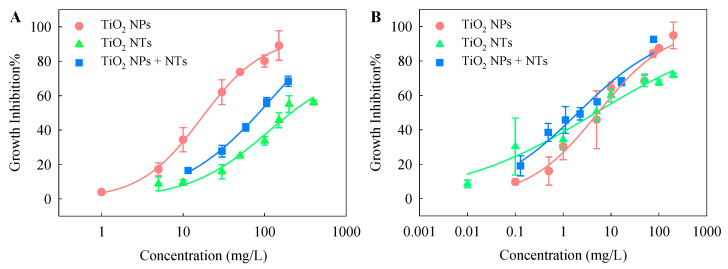
Concentration-response curves for *Scenedesmus obliquus* (**A**) and *Chlorella pyrenoidosa* (**B**) exposed to TiO_2_ NPs, TiO_2_ NTs, and TiO_2_ NPs + NTs.

Typical CRCs were observed for the toxic effects of TiO_2_ NPs, TiO_2_ NTs, and TiO_2_ NPs + NTs on the two test species (Figure 3). The effect concentrations determined by the CRCs are listed in Table 2. The CRC analysis indicated a concentration-dependent variation in the individual and combined toxic effects of TiO_2_ NPs and NTs. For *S. obliquus*, the CRC for TiO_2_ NPs was distant from the CRC for TiO_2_ NTs and started at lower concentrations (Figure 3A). Moreover, the *EC*_10_ and *EC*_50_ values of TiO_2_ NPs were lower than those of TiO_2_ NTs, suggesting that the single toxicity of TiO_2_ NPs to the algae was higher than TiO_2_ NTs. Some previous studies have also indicated that the shape of the ENPs is a significant factor in determining the potency and magnitude of the toxicity effect on organisms [29,30,31]. The CRC for TiO_2_ NPs + NTs was in between that for TiO_2_ NPs and that for TiO_2_ NTs. Similarly, the *EC*_50_ value derived from the CRC for TiO_2_ NPs + NTs was between the *EC*_50_ value of each components in the binary mixtures. As mentioned above, the stability of TiO_2_ NPs + NTs in the test medium at the initial time was also between that of TiO_2_ NPs and that of TiO_2_ NTs. The findings for the growth inhibition toxicity to *S. obliquus* combined with the findings for the stability indicate that the higher the initial stability, the stronger the toxicity. Dispersion of ENPs has received special research attention because the environmental behavior and effects of ENPs are greatly dependent on their dispersion status [32]. Previous studies have also suggested that nano-TiO_2_ aggregates can reduce the light available to the entrapped algal cells and thus inhibits their growth [33,34]. As mentioned above, the TiO_2_ particles agglomerated under this study. This also means that the agglomeration of particles contributed to the overall growth inhibition toxicity to some degree.

For *C. pyrenoidosa*, the CRC for TiO_2_ NPs crossed over the CRC for TiO_2_ NTs (Figure 3B). The *EC*_10_ value of TiO_2_ NPs was higher than that of TiO_2_ NTs. The *EC*_50_ value of TiO_2_ NPs was slightly higher than that of TiO_2_ NTs. However, as can be seen from the CRCs, the effect concentrations of TiO_2_ NPs were lower than those of TiO_2_ NTs, as the observed effects gradually increased. This means that the differences in the single toxicity of TiO_2_ NPs and NTs depend on the exposure concentration. Moreover, in the higher exposure concentration range, TiO_2_ NPs with the higher initial stability showed more toxicity than TiO_2_ NTs with the lower initial stability. The CRC for TiO_2_ NPs + NTs intercrossed the other two CRCs and decreased slightly with lower concentrations. The *EC*_50_ value derived from the CRC for TiO_2_ NPs + NTs was lower than that of TiO_2_ NPs and that of TiO_2_ NTs. This implies that the joint toxicity of TiO_2_ NPs and NTs to *C. pyrenoidosa* was higher than the single toxicity of each component in the binary mixtures.

It was also found that the *EC*_10_ and *EC*_50_ values of TiO_2_ NPs, TiO_2_ NTs, and TiO_2_ NPs + NTs to *C. pyrenoidosa* were lower than those to *S. obliquus*, which implies that the single and binary mixtures exhibited stronger toxicity to *C. pyrenoidosa* than to *S. obliquus*. This finding reveals that *C. pyrenoidosa* is more sensitive to the TiO_2_ particles than *S. obliquus*. *S. obliquus* is usually composed of four cells that are 12–34 μm wide and 10–21 μm long, with a flat shape. *C. pyrenoidosa* has a spherical shape with a diameter of 3–5 μm. The cells of *C. pyrenoidosa* are smaller but have a higher specific surface area than those of *S. obliquus*. This feature allows for a more effective particle uptake by *C. pyrenoidosa*. Furthermore, our previous study indicated the cell membrane permeability of *C. pyrenoidosa* was significantly increased after ENP stimulation, compared with the control and *S. obliquus* [35]. Consequently, the TiO_2_ particles might interrupt the cell membrane functions of *C. pyrenoidosa* to a higher degree, and thus trigger more severe growth inhibition toxicity.

### 3.3. Assessment and Prediction of Joint Toxicity of TiO_2_ NPs and NTs

**Figure 4:**
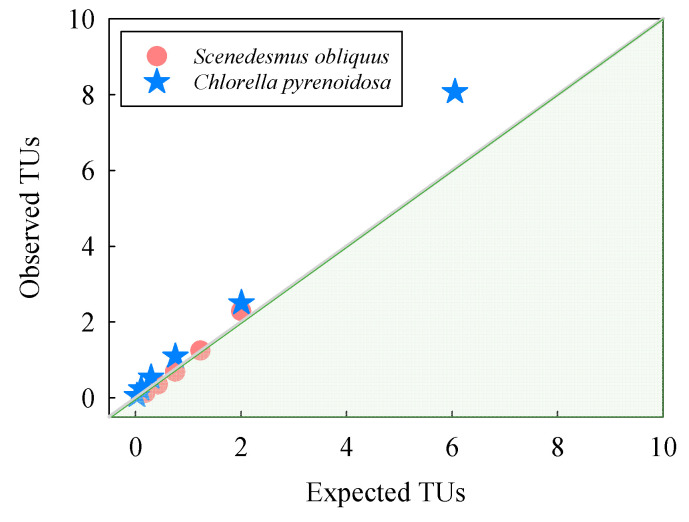
Observed toxic units (TUs) as converted from the toxicity data of *Scenedesmus obliquus* and *Chlorella pyrenoidosa* following exposure to mixtures of TiO_2_ NPs and NTs, and subsequently plotted against expected TUs calculated on the basis of the median effect concentrations from the individual constituents present in the mixtures.

The observed toxicities were converted to TUs and plotted against the expected TU_total_ values of the binary mixtures of TiO_2_ NPs and NTs, as calculated from the sums of the individual TiO_2_ NPs and NTs. As depicted in Figure 4, the observed TU_total,obs_ are almost equal to the expected TUs, indicating that the TiO_2_ NPs + NTs mixture effects were additive. However, for *C. pyrenoidosa*, the observed TU_total,obs_ of TiO_2_ NPs + NTs at the highest concentration under this study was obviously higher than the expected TUs, indicating that the joint toxicity was synergistic at higher concentrations of the mixtures.

**Figure 5:**
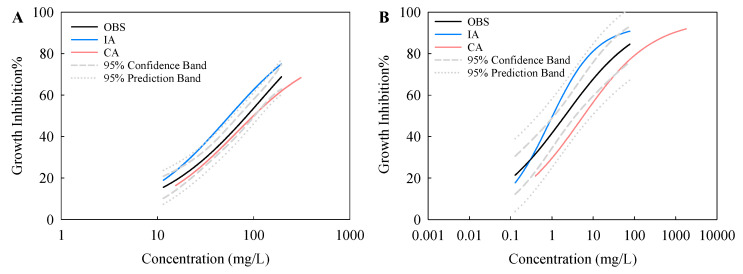
Comparison between regression models of the observed (OBS) combined nanotoxicity and expected toxicity of the mixtures to *Scenedesmus obliquus* (**A**) and *Chlorella pyrenoidosa* (**B**) according to concentration addition (CA) and independent action (IA). The dashed-line and dotted-line represent the 95% confidence band and 95% prediction band of the experimental data points, respectively.

The classical methods, namely, CA and IA, were used to quantitatively assess and predict the combined effects of ENPs [36,37]. The differences between the experimental and predicted joint toxicities to *S. obliquus* and *C. pyrenoidosa* are shown in Figure 5 and Table 2. For *S. obliquus*, the CRC derived from the CA model slightly deviated from the observed CRC, while the CRC derived from the IA model seriously deviated from the observed CRC (Figure 5A). For a direct graphical assessment of the whole concentration-response range, we also depicted the 95% confidence band (CB) and prediction band (PB) of the experimental data points. The CA prediction almost overlapped with the CB of the observed concentration-response data, implying that the CA model showed good predictive quality over the widest range of effects. However, except for the lower effect regions (about <40%), the IA prediction was outside the PB range of the observed concentration-response data. This also means that there was a big difference between the observation and the prediction for the IA model. As can be seen from Table 2, the *EC*_50_ value predicted by the CA model (99.46 mg/L) approaches the observed *EC*_50_ (85.04 mg/L), and the differences in the *EC*_50_ value between the observed and the CA predicted is 17%. However, the differences in the *EC*_50_ value between the observed and the CA predicted (56.30 mg/L) is 34%. Generally, the CA model performed better than the IA method although the CA slightly underestimated the observed toxicity of the binary mixtures of TiO_2_ NPs and TiO_2_ NTs to *S. obliquus*. This might be because the predictive power of the CA model was strictly restricted by the concentration addition MOJA of TiO_2_ NPs and TiO_2_ NTs to *S. obliquus*.

For *C. pyrenoidosa*, the CRC derived from the CA and IA models deviated moderately from the observed CRC (Figure 5B). Moreover, the concentration-response data predicted by the CA and IA models were inside the PB range of the observed data. Further, in the higher effect regions (about >65%), the CA prediction was inside the CB range of observed concentration-response data. Except for the range of effects from about 50% to 90%, the IA prediction was inside the CB range of observed concentration-response data. As shown in Table 2, the *EC*_50_ value predicted by the CA model was 2.9 times greater than the observed *EC*_50_ value. However, the *EC*_50_ value predicted by the IA model was 1.9 times lower than the observed *EC*_50_ value. Similar to *S. obliquus*, the CA underestimated the joint toxicity, while the IA model overestimated joint toxicity to *C. pyrenoidosa*. Generally, the IA model performs better than the CA method. This further implies that the MOJA of TiO_2_ NPs and TiO_2_ NTs to *C. pyrenoidosa* is mainly based on response addition. Taken together, the CA and IA methods provided valid predictions of the toxicity of the mixtures.

### 3.4. Cellular Oxidative Stress Effects of Single and Mixtures of TiO_2_ NPs and NTs on Algal Cells

**Figure 6:**
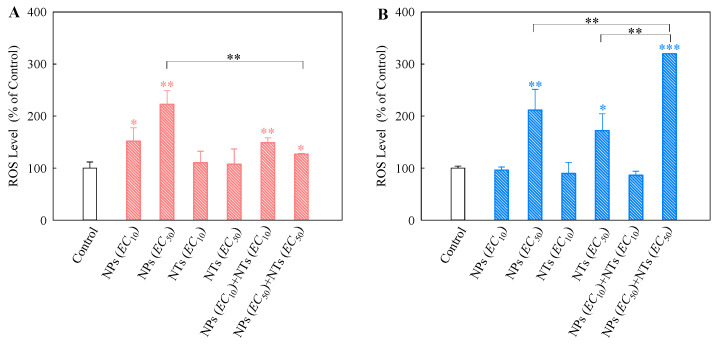
Relative levels of reactive oxygen species (ROS) detected using 2′,7′-dichlorodihydrofluorescein diacetate (DCFH-DA) staining in *Scenedesmus obliquus* (**A**) and *Chlorella pyrenoidosa* (**B**) exposed to single TiO_2_ NPs and TiO_2_ NTs at each *EC*_10_ or *EC*_50_ value, as well as TiO_2_ NPs + NTs at the *EC*_10_ or *EC*_50_ ratios. Statistical significance versus control group: * *p* < 0.05, ** *p* < 0.01, and *** *p* < 0.001.

Cellular oxidative stress caused by the elevation of particle-induced ROS is considered the most likely toxic mechanism of nano-TiO_2_ [38,39,40]. As shown in Figure 6A for *S. obliquus*, the FI (%) of the TiO_2_ NPs is significantly higher (*p* < 0.05) than the control, which indicates a significant increase in ROS. However, there was no significant difference in the ROS level between the TiO_2_ NTs and control. This implies that TiO_2_ NPs, but not TiO_2_ NTs produce ROS in *S. obliquus* cells. The binary systems of TiO_2_ NPs and NTs significantly promoted the generation of intracellular ROS. Note that the ROS levels induced by the binary TiO_2_ NPs + NTs mixtures at the *EC*_50_ ratio were significantly lower than the ROS levels induced by single TiO_2_ NPs at the *EC*_50_ value.

For *C. pyrenoidosa*, the TiO_2_ NPs and NTs at their *EC*_50_ value, as well as the TiO_2_ NPs + NTs at the *EC*_50_ ratio significantly increased the ROS levels (Figure 6B). Furthermore, the binary mixtures of TiO_2_ NPs and NTs induced the generation of intracellular ROS to a higher level than the single TiO_2_ NPs and NTs, which may intensify the oxidative stress effects on the *C. pyrenoidosa* cells exposed to the combination of TiO_2_ NPs + NTs. This also causes more serious apparent toxicity, as we observed in the growth inhibition toxicity testing. In general, TiO_2_ particle-induced ROS production depended on the particle characteristics, algal cell types, and exposure concentrations. In addition to the mechanisms underlying ROS generation, it also remains unknown as to how the TiO_2_ NPs interact with the TiO_2_ NTs and how this interaction regulates the intracellular ROS levels. Nano-TiO_2_ can cause genotoxicity [41]. Furthermore, the ROS-mediated stress within cells could be the main mechanism for the genotoxicity of nano-TiO_2_ [42]. Further studies are needed to explore whether the TiO_2_ NPs and NTs can jointly cause DNA damage due to the production of ROS.

## 4. Conclusions

To sum up, for the first time we present the toxicity of multiple ENP systems with different dimensions. It was found that the single toxicity varied as a function of the TiO_2_ dimensions, the test species, and exposure concentrations. The toxicity of the binary mixtures of TiO_2_ NPs (zero-dimension) and NTs (one-dimension) to two freshwater algae was found to be an additive joint activity according to the TUs. The classical toxicological models (CA and IA) for mixtures predicted the joint toxicities and revealed that the TiO_2_ NPs and NTs acted as a concentration addition and response addition towards *S. obliquus* and *C. pyrenoidosa*, respectively. The mechanisms of TiO_2_ NPs-NTs joint toxicity were related to the aqueous stability of the TiO_2_ particles and their ROS-induced oxidative stress effects. Our findings highlight the importance of the dimensions of nanoparticles in assessing the combined risks of multiple ENPs.

## Figures and Tables

**Table 1:** Zeta potential (*ZP*) and hydrodynamic diameter (*HD*) ± standard deviation (*n* = 3) of the test materials from single to binary mixture systems.

Test Materials	0 h		96 h	
	*ZP* (mV)	*HD* (nm)	*ZP* (mV)	*HD* (nm)
TiO_2_ NPs	−14.4 ± 0.2	969 ± 357	−13.2 ± 0.3	643 ± 84.5
TiO_2_ NTs	−12.5 ± 1.0	748 ± 201	−13.7 ± 2.3	681 ± 94.5
TiO_2_ NPs + NTs	−12.8 ± 0.5	943 ± 99	−12.9 ± 0.4	477 ± 21.1

**Table 2:** Effect concentrations derived from concentration-response curves of the single nanoparticles and the mixtures *^a^*.

Materials		*Scenedesmus obliquus*		*Chlorella pyrenoidosa*	
*Single Toxicity*		*EC*_10_ (mg/L)	*EC*_50_ (mg/L)	*EC*_10_ (mg/L)	*EC*_50_ (mg/L)
TiO_2_ NPs		2.33	19.75	0.13	5.38
		[1.75–3.44]	[17.02–23.00]	[0.11–0.14]	[3.36–8.23]
TiO_2_ NTs		13.16	211.26	0.002	4.87
		[5.08–25.75]	[152.01–427.73]	[0.002–0.004]	[2.81–9.66]
*Combined Toxicity*		*EC*_50_ (mg/L)		*EC*_50_ (mg/L)	
TiO_2_ NPs + NTs	OBS	85.04 [69.50–102.54]		2.05 [1.16–3.88]	
	IA	56.30 [56.17–56.42]		1.08 [0.91–1.34]	
	CA	99.46 [99.09–99.81]		5.96 [4.97–7.36]	

*^a^ EC*_10_*/EC*_50_ = 10%/mean effect concentration. The 95% two-sided confidence interval is shown in []; OBS = observed combined toxicity; IA = independent action; CA = concentration addition.
